# Morgan County Medical Society

**Published:** 1867-01

**Authors:** R. E. McVey, C. T. Wilbur

**Affiliations:** Pres’t.; Sec’y.


### MORGAN COUNTY MEDICAL SOCIETY.

Thursday, December 11th, 11 o'clock, A.M. Society met pursuant to adjournment.

Dr. Wagely was elected to preside, in the absence of the President and Vice-President.

The Secretary read the minutes of the last meeting, which were adopted.

The President called for the report of committees.

Committee on examination of credentials reported unanimously in favor of Dr. Dutton for membership, who was immediately elected by unanimous vote.

Dr. Prince exhibited several varieties of instruments for producing spray for medication of the air-passages, and for deadening the sensibilities of parts in order to lessen or prevent the pain attendant upon surgical or dental operations. He went briefly into a history of the introduction of the method of inhaling medical substances blown off in spray, as first practiced in France and Germany, and then explained the instrument invented by Dr. Richardson, of London, for the production of insensibility of parts by blowing upon them a spray of ether or rhigoline, which is capable of freezing in a few seconds by the tendency of rapid evaporation to produce cold. He exhibited an ingenious modification of the apparatus devised by Dr. Black for the purpose of shielding the lips and tongue from the spray while it is being blown upon the gums preparatory to extracting teeth.

Dr. Prince referred to cases in which union by the first intention had occured under unfavorable circumstances after the application of the spray of ether to the cut surfaces. He thought this result was secured by the speedy arrest of the flow of blood from the minute vessels under the influence of cold.

Dr. Edgar confirmed this view, by selecting cases occuring in connection with the battles at Vicksburg, in which union by the first intention had been secured by the application of ice to the wounds immediately after the amputation and before the final dressing.

Dr. Black made some remarks explanatory of his employment of the spray in extracting teeth, thinking the instrument a valuable means of lessening or destroying sensibility. In some cases he had found patients unable to bear the sudden reduction of the temperature on account of the exposed and irritable condition of the nerve of the tooth. In some of these cases he had succeeded in employing the spray by first covering the tooth with wax.

On motion it was resolved that the thanks of the Society be tendered Dr. Black for his valuable contribution to dental surgery.

The subject of local anæsthesia being under consideration, Dr. Edgar related a case of anchylosis of the elbow-joint of a young lady, treated with chloroform and olive oil to the joint, enclosed in oil silk to prevent evaporation, by which means all sensation was removed, the adhesions were broken up and the joint restored.

Society adjourned to meet at 2 P.M.

At 2 o'clock P.M., the Society met. The Vice-President in the chair.

Dr. Warriner presented samples of his purified castor oil to the society for inspection and trial.

The Vice-President reported a novel and interesting case of menstruation and escape of liquoramnii during eight months of pregnancy.

Dr. Wagely moved the subject of puerperal fever be taken up.

Dr. Prince apologized for the delay of his paper on that subject, and promised it should be forthcoming at the next meeting.

Dr. Edgar called attention to the liability of error in practice from failure to diagnose correctly the pathological condition in each case of puerperal fever, *i.e.*, whether dependent on a true toxæmia, or on metritis, phlebitis, peritonitis or metroperitonitis. That the toxæmic condition being attended with an asthenic grade a fever indicated a supporting treatment while the purely inflammatory cases might be better treated by early free depletion and full exhibition of anodynes.

The further consideration of the subject was postponed until the next meeting.

On motion the Society adjourned to meet on the second Thursday of January, 1867, at 1 o'clock P.M.

R. E. McVEY, M.D., *Pres't.*

C. T. WILBUR, M.D., *Sec'y.*

